# Supplementary material for: Are carbon emissions trading and green financial instruments synergistic? -Comprehensive quantitative research based on content analysis
Source: PLoS One. 2024 Mar 7;19(3):e0298601. doi: 10.1371/journal.pone.0298601 (PMC10919601; doi:10.1371/journal.pone.0298601)
Supplement: S1 Data — (ZIP) [file pone.0298601.s001.zip › Data/地级市Z_F工作报告.zip!/╣π╢1⁄2/╒┐╜¡╩╨/└┤╫╘╣1⁄2╓┌║┼í╛┬φ┐╦╩2╛▌═°í┐_2017.docx]

各位代表：

　　现在，我代表市人民政府向大会报告工作，请予审议，并请市政协各位委员和其他列席人员提出意见。

2016年及本届政府任期工作回顾

　　2016年，是湛江“十三五”良好开局之年。我们在中共湛江市委的领导下，全面贯彻党的十八大和十八届三中、四中、五中、六中全会精神，深入贯彻习近平总书记系列重要讲话精神，认真贯彻省委、省政府粤东西北振兴发展战略，认真落实市委决策部署，主动适应发展新常态，牢固树立新发展理念，聚力“三大抓手”，坚守“两条底线”，敢于担当，实干作为，实现经济运行稳中有进，保持经济社会持续健康发展，较好完成市第十三届人大六次会议确定的目标任务。

　　初步预计，全市生产总值2570亿元，增长8.2%。三次产业结构比19.1∶38.1∶42.8。工业总产值2860亿元，增长14.4%。固定资产投资1550亿元，增长18%，其中180项省市重点项目完成投资366.7亿元。外贸进出口总额305亿元，其中出口总额195亿元，增长11.9%。社会消费品零售总额1430亿元，增长9.5%。来源于湛江的财政总收入466.23亿元，一般公共预算收入112.97亿元。金融机构本外币各项存款余额2847.3亿元，贷款余额1633.8亿元，存贷比57.4%。居民人均可支配收入17928.9元，增长7.8%。

　　过去一年，中科炼化一体化项目全面动工，湛江成为我国新兴的现代钢铁产业基地，成为首批国家海洋经济创新发展示范城市和联合国可持续发展先锋城市。国家编制北部湾城市群发展规划，支持湛江建设区域中心城市。成功举办2016中国海博会、北部湾经合组织第九次成员大会，发起成立北部湾城市合作组织，全面启动琼州海峡港航一体化发展，全国沿海沿边地区基层反走私综合治理现场会在我市召开，湛江中心城市美誉度进一步提升。

　　一年来，我们主要做了以下工作：

　　（一）坚定改革开放、创新驱动，发展活力有效释放。改革攻坚有新突破。深入推进“放管服”改革，公布市直单位权责清单，取消、降低159项行政事业性和小微企业项目收费，赋予海东新区24项市级行政许可事项，向市和区级工业园区下放6类行政审批事项。商事制度改革深入推进，商事登记与开办企业便利度分别排名全省第三和第五位。深化普惠金融改革，首家政策性融资担保公司开业运营。民营经济焕发新活力。召开民营经济工作千人大会，出台40条鼓励和支持民营经济发展政策措施，开展“暖企服务月”活动。民营经济增加值1600亿元，增长10%；民间投资800亿元，增长30%；新增市场主体3.46万户、注册资本335.3亿元，新增“个转企”1220家、“企上规”200家。创新发展能力有新提升。申报国家级高新区通过科技部专家评审。“南方海谷”总部和科技创新大厦基本建成，首批11个单位签约入驻。新增高新技术企业39家。众创空间和科技企业孵化器达到10家。驻湛高校、科研院所155项科研成果与地方企业成功对接。成功举办首届湛江（中国）创意设计大会和第二届“南方海谷杯”创新创业大赛。开放发展迈上新台阶。跻身“中国外贸百强城市”。2016中国海博会首邀泰国设立主宾国，国内外3100多家企业参展参会。水博会、东盟农博会和湛江海洋周成功举办。湛江保税物流中心封关运作，国家进境水果指定口岸投入营运，霞山江南果蔬批发市场建成开业。启用智能化线上自助通关，实现企业自助申报、自主纳税、自动放行。对外投资2.35亿美元，增长4倍。

　　（二）坚定推进供给侧结构性改革，产业结构调整、转型升级步伐加快。“三去一降一补”有序开展。71家国有关停企业实现市场出清，12家国有特困企业脱困，淘汰落后小造纸产能1.82万吨；商品房去库存49.5万平方米；落实“营改增”和降费政策，下调部分社会保险费率，推进电力、价格等市场化改革，企业减负51.2亿元；28项农村电网、交通、供水等补短板重大工程启动实施。

　　现代临港工业格局加快形成。湛江钢铁一期工程全面建成试产，完成钢产量545万吨，实现产值155亿元，成为我市第4个产值超百亿元工业企业。钢铁配套园区26个项目入园，盛宝科技、沪湛辅料等18个项目建成投产。石化园区入园项目10个。湛江晨鸣实现产值150.8亿元，增长22.6%，四期建成投产。大唐雷州电厂主体工程完成投资30亿元，占总投资的31%。产业集聚发展势头良好，全市产业园区签约项目83个，落地68个，投产35个，实现规上工业总产值1378.3亿元，增长16.7%；增加值385亿元，增长15.4%。湛江产转园规上工业总产值、增加值分别增长36%和21.8%。奋勇高新区17个项目动工，9个项目建成。

　　现代服务业升级发展。深入开展“十大扩消费行动”和质量提升行动。电子商务交易额705亿元，增长43%。成功拓展东北旅游市场，接待东北旅游团122个、游客1.17万人，“冬休来湛江”实现良好开局；金沙湾滨海休闲旅游区、鹤地水库分别成为国家4A景区和水利风景区；全市旅游接待、旅游收入分别增长13.7%和28.6%。港口物流快速发展，全市港口吞吐量2.56亿吨，增长16.2%，其中湛江港集团吞吐量1.13亿吨，增长24.5%。民航旅客吞吐量155万人次，增长29%。商品房销售额264.2亿元，销售面积446.1万平方米，分别增长31%和27.8%。体育、家政、养老服务蓬勃兴起，成为新的消费热点。

　　现代农业稳健发展。制定实施雷州半岛现代农业发展、水利综合治理和生态修复三大规划。正大（湛江）现代农业3000万只肉鸡养殖项目建成，12万吨饲料项目投产。广东北部湾农产品流通综合示范园区一期投入运营。湖光农场纳入西城新区整体规划，5个镇场融合发展，垦地共建雷州半岛现代农业示范核心区。建立台风巨灾保险制度，开展政策性岭南特色水果种植保险。建成农田配套渠系322公里、高标准基本农田188万亩，新增粮食储备18万吨。加固海堤254公里，完成153宗中小型病险水库除险加固。新建200吨以上钢质渔船72艘，累计达到162艘；5个市级区域性避风塘建成使用。

　　（三）坚定推进扩容提质，城镇发展有新变化。“三环四通”格局加快形成。交通建设完成投资88.1亿元。广湛高速官渡（互通）立交出口（二期）建成通车。东海岛铁路、黎湛铁路电气化改造完成投资过半；汕湛高速湛江段累计完成投资31亿元，占总投资42.7%。东雷高速全线施工。合湛高铁、玉湛高速、西城快线、高铁西站及其配套工程和调顺跨海大桥开工，湛江国际机场启动初勘与测量工作。徐闻港南山客货滚装码头、进港公路改扩建和湛徐高速徐闻港支线三大工程加快推进。湛江港宝满港区集装箱码头一期、30万吨级航道和湛江钢铁基地30万吨级码头通过验收，东海岛港区公共深水码头动工建设。改造国省道61.8公里，累计改造716.4公里，完成国省道“迎国检”任务；改造县道76.5公里，如期实现“3年改造300公里县道”目标；完成村道硬底化633公里。

　　城市扩容提质扎实推进。编制完成新型城镇化和低碳生态城市建设规划。主城区完成赤坎“三民”路修缮改造，实现霞海码头功能腾退；广州湾大道南段全线贯通，体育北路西段等3条市政道路建成通车，世贸人行天桥动工，源珠路、南方路、海湾路等市政道路加快建设；建成滨湖公园12.6公里环湖绿道，银帆公园基本建成；数字化城管中心上线运行；强化“六乱”“两违”整治，拆除违法建设22.9万平方米，市区违法建设保持“零增量”；完成“三旧”改造1360亩。新城区建设有序展开，海东新区建成东城西路等9条道路，25公里海东快线全线通车；军港大道、湛江实验小学等15个项目动工；地下综合管廊项目获得国家2.3亿元专项建设基金；完成三柏西等3条村庄整村搬迁，两条村农民公寓竣工。西城新区编制完成教育城、高铁新城控制性详细规划。东海岛中央商务区和千亩生态公园动工建设。

　　县城建设与县域经济发展开启新局面。五个县城完成26条86公里道路“白改黑”，雷州建成人民公园和9.5公里西湖水库绿道，廉江建成8.4公里北部湾大道二期工程，吴川沿江大道、徐闻徐湛路改造竣工，遂溪建成商业新城和孔子文化城等项目。预计县域生产总值、固定资产投资分别增长9%和25%。

　　（四）坚定推进绿色发展，生态文明建设取得新成效。“三个城市”建设有效推动城市转型。脚印城市建设成为2016年中国城市化优秀案例，320辆新能源公交车投入使用，市区新开公交线路12条，中心城区公共自行车租赁点实现全覆盖。建成万达广场、教育城等海绵城市示范小区。钢铁、炼化两大项目开展循环经济取得突破，奋勇高新区成为省级循环经济示范园。赤坎金沙湾“三个城市”样板区建设成效显著，垃圾“五分”深受欢迎，实现分类回收和集中处理。

　　生态环境整治与保护力度加大。PM2.5年均值降至26微克/立方米，城市环境空气质量优良率97%；城市供水水源和近岸海域环境功能区水质100%达标。主城区治水持续推进，建成绿塘河污水提升泵站，完成百蓬路和友谊路“水浸街”黑点整治。九洲江-鹤地水库流域治理纳入国家“水十条”项目储备库，小东江流域环境整治扎实推进。市区、廉江生活垃圾焚烧发电厂和徐闻、遂溪、吴川生活垃圾填埋场投入运营，赤坎水质净化厂三期基本建成，黄坡等中心镇生活污水处理设施建成使用。淘汰黄标车13749辆。更新造林10.3万亩，营建生态林3.05万亩。

　　（五）坚定推进共享发展，社会民生持续改善。民生保障更加有力。市财政社会民生投入308.67亿元，占公共预算支出80.5%。十件民生实事基本完成，十大民心工程推进实施。新增城镇就业7.8万人、失业人员再就业3.7万人。市养老服务中心基本建成，企业职工养老保险待遇提高6.5%，基本医疗保险覆盖率98.8%，22100多名市直国资系统困难企业退休人员基本医疗保险问题得到妥善解决。省内异地联网结算定点医院增加到131家。与13个省内外城市建立公积金合作关系。城乡居民低保月标准分别提高到490元和340元。建成保障房1572套，完成危房改造19515户。各级财政投入扶贫资金9.46亿元，实施精准扶贫项目12161个，74000多名扶贫对象如期脱贫。解决9个县（市、区）2000多条自然村150万群众饮水安全问题。

　　社会事业有新进步。湛江中心人民医院新院主体基本建成，市区新增床位1196张；建成114家公建民营村级卫生站；县镇医疗卫生服务管理一体化改革全覆盖，14间县级公立医院直管87间乡镇卫生院。市区新建扩建中小学校6所，新增学位10500个；完成18所薄弱普通高中改造。教育城海大寸金学院新校区和湛江幼儿师专一期建成使用，驻湛普通高校达到6所。湛江籍运动员李丽青勇夺第15届里约残奥会盲人柔道女子48公斤级冠军。成功举办全国水球、手球锦标赛和北部湾城市网球邀请赛，列为省足球试点城市。基层公共文化设施实现全覆盖。湛江作家黄康生荣获“第七届冰心散文奖”。湛江云媒成功上线。遂溪林雪平家庭被评为第一届“全国文明家庭”，雷州村民麦家福被评为“中国好人”。全面落实“两孩”政策，人口自然增长率11.37‰。外事侨务、港澳台、民族宗教、气象地震、人防海防、社会科学、妇女儿童、残疾人、地方志、老区等工作进一步加强。

　　社会大局和谐稳定。深入开展“飓风2016”等专项行动，完善社会治安防控体系，推进创建“平安细胞”，全市违法犯罪警情下降1.9%，市区“两抢”警情下降21.5%。建成121个镇街社区戒毒康复工作站，雷州禁毒重点地区整治成效明显。被评为全国“六五”法治宣传教育先进城市。严格落实安全生产责任，连续8年没有发生重特大生产安全事故。“明厨亮灶”与网格化食品药品安全监管经验分别在全国全省推广。移动应急指挥平台、主城区应急供水系统建成使用。信访渠道进一步畅通，积案化解成效明显。麻章区行政村基层组织重构试点走在全国前列。建立军地深度融合发展机制，军政军民关系团结融洽，第五次荣获“全国双拥模范城”、第六次荣获“省双拥模范城”称号。

　　（六）深入践行“两学一做”，政府建设进一步加强。深入开展“两学一做”学习教育，全面落实中央八项规定精神，坚决反对“四风”，市本级“三公”经费支出下降32.9%。建立健全政府重大事项集体决策、专家咨询、社会公示制度，完善政府法律顾问机构。主动接受人大依法监督和政协民主监督，办理人大代表建议、政协委员提案250件，办复率100%。网上办事分厅APP手机版成功上线，120个镇（街）、1778个村（社区）网上办事站（点）开通，基层公共服务平台基本建成。12345市民服务热线荣获“全国三八红旗集体”称号。市政府门户网站浏览量近4000万人次，微信公众号阅读量73.6万人次，获得2016年度中国政务网站领先奖。

　　各位代表：过去五年，我们坚持在振兴发展中勤勉履职，在攻坚克难中探索创新，在重重考验中稳步前行，有效应对经济下行压力，战胜“彩虹”等超强台风和严重干旱灾害，主要经济指标稳步增长，建成一批事关全局的重大项目，办成一批惠民利民的好事实事。五年来，我们坚持发展第一，着力培育现代产业，聚精会神建园区抓招商上项目，持续推动钢铁、石化、纸业等临港产业项目建成投产或全面开工，挺起湛江现代工业的脊梁；持续开展产业园区建设与招商引资“三讲三评”，支持园区完善设施、引进项目、扩能增效；持续改善政务、诚信、治安和交通四大投资环境，推进实施“十百千”投资计划，出台优惠政策大力发展民营经济，鼓励乡贤回归；持续推进县域工业化、城镇化，支持县城扩容提质，推动县域工业发展；持续壮大商贸物流园区，建设“五岛一湾”滨海旅游产业园，引领服务业升级发展；持续推进现代农业示范区建设，绿色食品、农业品牌数量位居全省前列。坚持交通先行，着力推进“三环四通”，先后建成茂湛铁路、东海岛疏港公路和湖光、雷湖、海东快线，总长151公里的环城高速基本成形；持续推进深水航道和集装箱、大宗散货码头建设，实施“一县一港、一港一园”战略，实施航空“百万工程”，有效推动资源要素集中、人流物流集聚，区域中心城市影响力持续提升。坚持扩容提质，着力推动城市转型发展，脚印城市、海绵城市和循环城市“三个城市”理念普及落地，海东新区开发全面启动，海西完成市政干道改扩建、“白改黑”，“六乱”“两违”整治初步告捷，中心城区在有序开发中扩容，在转型发展中提质，实现了城市升级、格局大变。坚持改革开放、创新驱动，着力增强发展活力，持续推进重点领域和关键环节改革，推动军地、校地（院地）和垦地“三地”融合发展，建设创新创业平台，推动形成“双创”热潮；积极融入北部湾城市群和琼州海峡港航一体化发展，坚持双向开放首选东盟，湛江口岸与东盟双边贸易增长7倍，成为“一带一路”海上合作战略支点城市。坚持守住底线，着力实施蓝色崛起与蓝天碧水“双蓝”战略，实施主城区治水工程，攻坚完成港湾清障和南柳河整治，建成两个海滨浴场和瑞云湖、滨湖公园，市区重点河渠截污纳管，鹤地水库水质明显改善，城市环境空气质量保持全国前列、全省领先，实现了发展与美丽共赢。坚持民生为重，着力推动共建共享，先后办成一批民生实事、建成一批民心工程，圆满完成扶贫开发“双到”任务，医保“湛江模式”全国推广，医改“湛江模式”成效明显，社会保障更加健全、公共服务更加优质、社会大局更加稳定。

　　各位代表！历经五年的拼搏奋斗，我们深感湛江振兴任重道远，推动湛江振兴务必勇于进，坚持发展第一要务，聚焦“三大抓手”“两条底线”，精准发力、久久为功，稳中求进、加快发展。务必敢于闯，依靠改革破除藩篱、清除障碍，有效凝聚发展共识；依靠创新厚植优势、驱动发展，有效汇集要素资源，实现跨越发展。务必顺于势，坚定对接国家和省发展战略，主动融入经济全球化、区域经济一体化，推动开放发展；坚持区域协调、以市带县，实现均衡发展。务必敬于民，坚持以人民为中心，“为民而建”“为用而建”，尊重民意、改善民生，保障民利、惠民发展。务必勤于干，坚持把“干”作为一种责任、一个追求、一份光荣，勤勉努力、敢于担当，主动作为、实干发展。务必严于纪，严守纪律、严守规矩、严守法律，不忘初心、继续前进，亲而又清、干净干事，清廉用权、清正发展。

　　各位代表，五年风雨路，团结谱新篇。来之不易的发展成就，是市委坚强领导的结果，是全市干部群众团结拼搏的结果。我代表市人民政府，向全市人民，向市人大代表和市政协委员，向各民主党派、工商联、无党派人士、人民团体和社会各界人士，向中央和省驻湛单位，向驻湛解放军、武警部队官兵和政法干警，向所有关心支持湛江振兴发展的海内外友好人士，表示衷心的感谢和崇高的敬意！

　　回顾五年工作，问题必须正视。主要是：经济总量不大、综合实力不强，人均水平不高，落后欠发达的地位依然没有改变；经济结构不优，传统产业转型升级不快，新兴产业成长不足，应对下行压力、稳定增长预期的基础依然不牢；交通事业发展依然滞后，全市人民对尚未开通的高铁翘首以盼；城市管理依然比较粗放，精细化管理能力亟待提升；区域发展很不平衡，县域经济依然是全市协调发展的薄弱环节；社会民生短板较多，精准脱贫任务依然繁重；振兴发展与环境保护的双重考验依然严峻，人民对湛江永葆蓝天碧水高度关注；市民对政府公共服务满意度不高，少数政府工作人员改革创新、责任担当、服务保障和纪律法治意识欠缺，作风不正、为官不为、懒政怠政依然存在，有的重点项目、重点事项尤其是民生实事、民心工程进展不快，人民群众不够满意，消极腐败现象时有发生。对此，必须采取更加精准、扎实、有效的举措，坚决克服纠正。

新一届政府任期奋斗目标及2017年工作安排

　　各位代表！新起点开始新发展，新长征承载新使命。新一届政府任期肩负人民过上幸福小康生活的美好期待，肩负国家和省寄予湛江建设区域中心城市的厚望重托。根据市第十一次党代会精神，未来五年市政府工作总体要求是：高举中国特色社会主义伟大旗帜，以邓小平理论、“三个代表”重要思想、科学发展观为指导，深入学习贯彻习近平总书记系列重要讲话精神，全面贯彻落实“五位一体”总体布局、“四个全面”战略布局和“五大发展理念”，聚焦产业强市，推动港产城融合发展，坚持蓝色引领、“双化”驱动、市县并进、对接珠三角、联动北部湾，全力推进“三大抓手”，坚决守住“两条底线”，深化改革创新，着力改善民生，与全省同步全面建成小康社会，加快建设环北部湾中心城市。

　　主要奋斗目标是：经济实力更强。经济结构进一步优化，工业经济总量做大，农业现代化进程加快，服务业贡献率不断提高。地区生产总值年均增长10%左右，工业总产值年均增长13％以上，固定资产投资年均增长10％，社会消费品零售总额年均增长11%，外贸进出口总额年均增长7％，一般公共预算收入年均增长10%，居民人均可支配收入年均增长10％，产业结构调整为15∶44∶41。交通条件更捷。高速公路里程翻一番，高速铁路开通，湛江国际机场建成使用；“三环四通”港湾环、市区环基本形成，市县快速通道更加完善，重点中心镇基本通达一级公路，所有自然村实现道路硬底化；雷州半岛港口群初步形成，湛江港区域龙头大港地位更加巩固，区域性综合交通枢纽基本建成。城乡面貌更美。市区和县城扩容提质成效明显，公共服务设施不断完善，城市管理水平进一步提升。以人为核心的新型城镇化加快推进，城乡一体化进入新阶段。生态文明建设加快推进，继续保持良好的生态环境和人居环境，城市空气质量保持在全国环保重点城市前列。发展动力更足。重要领域和关键环节改革取得决定性成果，法治化、国际化、便利化的营商环境初步形成。“一带一路”战略深入实施，开放型经济发展水平不断提升。“大众创业、万众创新”蔚然成风，创新型城市建设取得明显进展。社会治理更好。依法治市全面推进，人民民主更加健全，司法公信力明显提高。“平安湛江”建设不断深化，社会更加和谐稳定。社会治理体系不断完善，治理能力进一步提高。社会主义核心价值观更加深入人心，创建全国文明城市取得显著成效。幸福指数更高。人民生活水平显著提高，精准扶贫任务圆满完成，全面建成小康社会。社会保障体系日趋完善，公共服务体系更加健全，精神文化生活更加丰富多彩，民生事业更加进步，人民群众幸福感获得感显著增强。政治生态更优。全面从严治党深入推进，“有激情、在状态、敢担当、善作为”的干事创业导向鲜明树立。党风廉政建设和反腐败斗争扎实推进，“两个责任”落实更加到位，标本兼治成效明显，干部作风持续改进，政治生态进一步净化。

　　2017年是新一届政府履职施政的开局之年。今年政府工作的基本思路是：全面贯彻党的十八大和十八届三中、四中、五中、六中全会精神，深入贯彻习近平总书记系列重要讲话精神，认真落实省委、省政府粤东西北振兴发展战略，认真落实市第十一次党代会精神，统筹推进“五位一体”总体布局，协调推进“四个全面”战略布局，主动适应发展新常态，自觉践行新发展理念，坚持稳中求进工作总基调，坚持把推进供给侧结构性改革作为经济发展的主线，聚焦“三大抓手”“两条底线”，全面做好稳增长、促改革、调结构、惠民生、防风险各项工作，着力构建创新型经济格局，着力振兴实体经济，促进经济平稳健康发展，促进社会和谐稳定，以优异成绩迎接党的十九大胜利召开。

　　今年经济社会发展的预期目标是：生产总值增长9%左右；规上工业增加值增长11%；固定资产投资增长12%；港口吞吐量2.65亿吨；社会消费品零售总额增长10%；居民消费价格指数涨幅控制在3%左右；外贸进出口增长7%；实际利用外资增长3.5%；一般公共预算收入可比增长10%；居民人均可支配收入增长9%；城镇登记失业率控制在3.5%以内；高中阶段教育毛入学率90%以上；常住人口自然增长率控制在11.7‰以内；城镇生活垃圾无害化处理率85%，完成省下达的节能减排降碳目标任务。

　　围绕上述目标，突出抓好以下工作：

　　（一）扩大需求提振信心，全力实现经济增长预期。加强引导形成良好发展预期，向市场释放积极信号，增强实体经济发展信心，全面汇聚稳定增长的正能量。

　　强化实体支撑大力发展民营经济。推动资源要素有效集聚，支持企业坚守主业、做强主业，促进实体经济提质增效。实施质量强市工程，弘扬开拓创新、执着敬业的企业家精神，弘扬精雕细琢、精益求精的工匠精神，扎实开展“增品种、提品质、创品牌”专项行动，推动内外销产品“同线同标同质”，支持企业锲而不舍提高产品、工程和服务质量，打造百年品牌、建设百年老店。振兴实体经济必须坚定“两个毫不动摇”，全面落实市发展民营经济“40条”，推动民营经济主体增加、投资增长、发展增效。建成中小（民营）企业服务中心，深入开展暖企服务，帮助企业解决历史遗留问题。推进投资准入负面清单改革，拆除显性和隐性门槛，引导民企、国企相互注资交叉参股，发展混合所有制经济；鼓励民营企业投资市政设施和公用事业，参与提供医疗、教育、养老等社会民生公共服务。用活用足省市中小企业发展专项资金，发挥政策性融资担保公司作用，支持民营企业加大创新投入，加快技术改造，提升企业素质，进一步加快“个转企”“企上规”步伐，培育造就一批民营大型骨干企业。

　　强化项目落地扩大有效投资。年内安排重点项目172项，计划投资382.6亿元。加强项目建设进度预警与协调处置，深化企业项目投资建设直接落地改革试点，推行重点项目建设多评合一、多审合一、多图联审、联合验收，坚决克服“前松后紧”现象，科学安排建设工期，确保签约项目快落地、动工项目快建设、建成项目快达产，加快形成实实在在的产能、就业和税收。注重发挥驻外商会的招商功能，深入实施“十百千”投资计划和“乡贤反哺工程”，支持新一代乡贤返湛投资兴业。坚持把招商引资作为干部锻炼成长主战场，选派年轻干部常驻广州、深圳、佛山、东莞等珠三角城市，面向国际国内500强企业“点对点”精准招商。加强PPP项目库建设，尽快推出一批政府与社会资本合作项目。

　　强化扶持引导释放消费潜力。全面实施“十大扩消费行动”，带动消费结构升级。实施“互联网+流通”行动计划，支持实体零售企业跨界融合、创新发展，通过线上线下互动，构建全渠道、跨领域、平台化发展新模式。落实电商物流与快递协同发展试点，改善农村消费环境。落实住房公积金普惠政策，满足干部职工合理的住房需求，培育旅游、养老、文化等地产新业态，促进房地产市场平稳健康发展。加快光纤入户，建设高速宽带网络，扩大信息消费。面向东北、华北、西北“三北”市场，开展“一路向南看海”等旅游主题促销，扩大“冬休来湛江”旅游消费。推动文化与旅游、科技、金融、新媒体融合发展，发展文化产业，扩大文化消费。实施促进体育消费行动计划，打造“健康之都”“运动之城”。拓展汽车消费，壮大休闲、养老、家政等服务业态，活跃节假日消费市场。

　　强化优进优出稳定外贸增长。支持企业用好用足出口退税、信保和便利通关等扶持政策，提升产品技术标准、品牌质量，发展在岸、离岸服务外包，建设跨境电商公共服务和境内外展示贸易平台，深耕传统市场，拓展东盟、南亚、中东、非洲等“一带一路”新兴市场，扩大装备产品和服务出口。支持企业申报进口贴息资金，扩大先进技术、重要装备和关键零部件进口。开辟更多外贸海运航线，大力发展集装箱、保税业务，加快东海岛、宝满口岸对外开放，建成国际贸易“单一窗口”电子口岸，启动申报综合保税港区。

　　（二）推进供给侧结构性改革，加快构建高水平产业体系。推进落实“三去一降一补”工作。积极淘汰落后产能，稳步推进国有企业出清重组和特困企业脱困。坚持去库存和促进人口城镇化相结合，着力化解商铺、写字楼和县域商品房库存。推动地方法人银行杠杆率达到4%的监管最低要求，有效降低企业负债率。推动降低各类交易成本特别是制度性交易成本，降低各类中介评估费用，降低企业用能、物流成本，增强劳动力市场的灵活性，支持企业降低综合成本。盘活财政积淀资金，增加建设资金有效供给，推进实施34项农村电网、天然气管网和信息基础设施等民生短板项目。

　　深入实施工业强基工程。落实先进装备制造业培育工作方案，促进工业化与信息化、制造业与互联网融合发展，壮大精品钢材、造船海工、机械装备、军工产品、生物医药等新兴产业；落实技改事后奖补政策，支持规上工业企业实施技术改造，推动家电、家具、水产加工、饲料、羽绒和制糖等传统产业智能化、绿色化发展。全力支持湛江钢铁基地达产达效，推动1550mm冷轧工程全线投入运营。实施“钢铁带动工程”，推动钢铁配套园区10个项目建成投产。加快中科炼化一体化项目主体工程建设，完成投资50亿元。启动实施湛江晨鸣五期。推进冠豪高新二期、中国纸业白卡纸、京信电力、鹏尊能源、大唐雷州电厂等重点项目加快建设。加快湛化集团搬迁，年内完成迁建投资3亿元。确定海滨船厂搬迁南三岛建设方案。

　　突出产业园区集聚发展。落实下放市直和区级工业园区的融资、项目、土地、创新、人才等管理权限，支持产业园区完善设施、引进项目、加快发展。加快建设钢铁、石化配套园区和中国物流湛江诚通综合物流园，推动湛江产转园扩能增效。鼓励赤坎、霞山、坡头、麻章和开发区建成区依靠创新驱动发展都市型工业，支持霞山华港工业小区发展高新技术产业。推动雷州市与奋勇高新区共融共建，发展食品医药、装备制造、电子电器和新材料产业。集聚造纸产业链项目，加快麻章森工产业园建设。支持廉江产转园建设全国小家电产业集群区域品牌试点。支持吴川产转园引入更多特色产业项目，规划建设湛江（吴川）空港经济区。支持建设徐闻特色农产品加工区，加快建设徐闻现代国际物流园。支持遂溪壮大食品产业，建设装备工业园。

　　升级发展现代服务业。加快建设中央商务区，推进城市核心商圈和特色商贸街区提档升级；建设湛江展览中心，办好中国海博会、水博会、东盟农博会和湛江海洋周，举办滨海旅游、家电、水产和珍珠专业展会，壮大会展产业。实施大数据战略，推动信息基础设施共建共享，加快超高速无线局域网在智慧交通、智慧城市、工业互联、城乡社区的推广应用，培育物联网、云计算产业，发展信息经济。建设文化创意产业园，发展都市文化经济。加强金融保险业招商，创新金融产品服务，壮大金融服务业。发展冷链产业，完善城乡配送体系，规划建设初级农产品田头冷库和冷链物流园区，提高农产品流通效率。支持湛江港建设区域性石油、铁矿石交易中心。加快实施滨海城市旅游“3844”工程，建设中国南方冬休基地和广东全域旅游示范区。有效整合海岛、海湾、海滩、海景、海风和海鲜优势资源，加快“五岛一湾”滨海旅游开发，建设特呈全域旅游岛和南三岛滨海旅游示范区，规划引导硇洲岛旅游发展，提升发展湖光岩大旅游圈和金沙湾休闲旅游区，推动建设滨海旅游风情小镇，拓展滨海特色、半岛人文旅游，发展休闲、养生、康体旅游，开发邮轮、游艇旅游。深化区域旅游合作，创建“国家诚信旅游示范城市”。

　　（三）坚持陆海空并进，加快构建区域性综合交通枢纽。实施重点交通建设项目25个，完成投资100亿元，继续推进“三环四通”大交通建设。建成东海岛铁路、汕湛高速湛江段以及高铁西站、西城快线；完成黎湛、河茂、茂湛铁路电气化改造，力争2018年一季度开通高铁。加快合湛高铁、东雷高速、玉湛高速、高铁西站客运综合交通枢纽和调顺跨海大桥等工程建设。推进湛江至徐闻高铁前期工作。全面完成征拆任务，加快湛江国际机场建设步伐。开工建设环城高速南三岛大桥、南三岛中线公路、汕湛高速吴川支线。升级改造省道290线，打造雷州半岛西部旅游公路。完成国道207线、325线遂溪段改线工程前期工作，力争开工建设廉湛快速路，推进雷州半岛环岛一级公路遂溪段和国道325线黄坡至梅菉段改扩建前期工作。加快实施徐闻港南山客货滚装码头、进港公路改扩建和湛徐高速徐闻港支线三大工程，启动湛徐高速乌石港支线、湛徐高速江洪港支线和渝湛高速龙头沙港支线以及粤海铁路乌石港支线前期研究工作。全力争取湛江港40万吨级航道列入国家规划，动工建设30万吨级双向航道、宝满港区集装箱码头两个改扩建工程和中科炼化配套码头，推进东海岛港区公共码头建设，落实东海岛、宝满港区配套铁路支线前期工作。推动实施“一县一港、一港一园”战略，加快徐闻、雷州、廉江和遂溪港区开发建设。

　　（四）坚持创新驱动发展，加快构建区域性科教创新中心。加快湖光创新创业“双创圈”规划建设。坚持蓝色引领、陆海统筹，加快实施21个重点项目，着力发展海洋生物、海洋装备产业，加快建设国家海洋经济创新发展示范城市。坚持岛区合一、产城融合，支持驻湛高校、科研院所、企业共建产业创新联盟，滚动实施重大基础研究、科技攻关和科技示范应用专项，重点建成“南方海谷”海洋产业孵化中心，推动建设教育城、高铁新城和湖光创新小镇，聚力建设湖光“双创圈”，加快建设创新型城市，形成区域创新高地，打造面向北部湾和东盟的海洋科技创新中心。实施高新技术企业培育工程，力争高新技术企业达到100家，争取获批国家高新区。加快建设“四众”平台。实施孵化器“倍增计划”，支持企业利用老旧厂房、闲置房屋发展新型众创空间，动工建设赤坎文化创意城。加快建设霞山智慧产业城。推广创意众包，引导企业通过网络向研发机构交付设计、研发任务。推动建设众扶平台，共助小微企业成长壮大。稳步推进实物、股权众筹，规范发展网络借贷。落实以增加知识价值为导向的分配政策，鼓励科研人员兼职创新创业。引入风险投资、天使投资和创业投资基金，壮大银行业科技支行、科技小额贷款公司，支持驻湛高校建设大学生创业中心、创业园，吸引更多创客扎根湛江。办好第二届湛江（中国）创意设计大会和“南方海谷杯”创新创业大赛。

　　（五）协调推进扩容提质，推动城市转型发展。着力推进城市治理方式创新。尊重城市发展规律，提升城市规划和设计水平。严格落实城市总体规划和土地利用规划，推进多规合一和“一张图”管理，建立城乡规划信息管理平台。加快智慧城市建设，整合数字化城管与交警智能指挥中心信息资源，强化综合执法与大数据监管，构建权责明晰、服务为先、管理优化、执法规范、安全有序的城市管理体制。建设市政路灯和城市排水防涝智能控制系统。落实市区主干道、人行道机械清扫保洁。有效遏制“六乱”，坚决整治“两违”，确保违法建设“零增量”。规范城市规划区村民建房管理，引导群众积极申报建房。加强市区电动自行车管理，推行合标电动自行车登记上牌，开展新一轮市区交通秩序整治。健全机制固化“创卫”成果，开展文明城市、县城、镇街、村居四级联创，提升城市文明水平。

　　着力推进海东开发。完成南调路升级改造、海东快线绿化亮化等工程；加快建设体育路等7条道路；动工扩建海川快线。建成5公里综合管廊和污水收集管网一期，推进实施污水处理厂等项目，动工建设海东公园。加快广东医科大学附属医院海东医院等重点项目建设，动工建设湛江文化中心。完成黄伍村整村搬迁，力争三柏西、力竹兜村农民公寓搬迁入住。

　　着力推进海西提质。完成南方路等9条道路改扩建，升级改造东新路、海湾路等10条道路，动工建设银帆路等6条主次干道。改造赤坎“三民”路外立面和霞山观海长廊、延安路片区，建设霞山法式风情街二期。实施23个“三旧”改造项目，启动改造旧大天然、三星和调顺岛片区，有序推进城市修补与更新工作。实施西城组团规划，有序推进高铁新城周边和湖光快线、西城快线、政通路沿线开发。

　　（六）坚持“以市带县”，推进区域协调发展。提升现代农业供给水平。深入推进农业供给侧结构性改革，深化农村综合改革，落实农村土地“三权”分置办法，加快农村土地承包经营确权登记颁证，开展农村土地承包经营权与农民住房财产权抵押贷款试点，赋予农民更加充分的财产权利。支持农垦深化管理体制等方面改革，推进垦地融合发展。加快实施雷州半岛农业、水利和生态修复三大规划。推动质量兴农，强化农产品标准化生产、品牌创建和质量安全监管，筹办热带水果展销会，有效增加特色水果、水产和北运蔬菜等绿色优质农产品供给。推进建设廉江、徐闻国家级现代农业示范区，加快建设雷州半岛现代农业示范核心区，发展都市休闲观光农业。支持徐闻县与海口市建立长期稳定的蔬菜购销供给机制。促进农村一二三产业融合发展，提高农产品精深加工和副产品综合利用率，积极创建省级农产品加工示范园区。培育发展农村电商、休闲农业、乡村旅游等新产业新业态。启动青年运河灌渠节水改造二期，实施水库扩容、水系联通联调，加快海堤加固达标、中小河流整治等工程建设。建设国省道绿化示范林180公里，重建沿海防护林带1.5万亩，营造碳汇林2.5万亩、热带季雨林5000亩、水源涵养林3000亩。加快建设硇洲中心渔港等6个渔港、避风塘，加快渔船升级改造，支持远海集约养殖，拓展“蓝色粮仓”，壮大远洋渔业。

　　做大做强县域特色经济。加快推进县域新型工业化、城镇化。鼓励各县（市）因地制宜发展优势主导产业，支持半岛北部的廉江、遂溪、吴川三县（市）做强家电、建材、食品、羽绒和滨海旅游产业；支持半岛南部的雷州、徐闻两县（市）立足特色资源，发展农海产品加工业和新能源、临港工业以及港口物流业，发展冬休度假、生态旅游、休闲养生、农业观光和历史人文旅游产业。支持县城干道改扩建，完善县城水电燃气、通讯网络和生活垃圾、污水处理设施，推动教育、医疗等公共服务资源向县城和中心镇配置，推进县城扩容提质发展。支持中心镇盘活资源、引进项目，建设特色鲜明、产城融合的农业强镇、工业大镇、商贸重镇和旅游名镇。坚持户籍证、居住证“两证并行”，鼓励农业人口向县城、中心镇转移。

　　（七）深化改革扩大开放，进一步增强发展活力。深化重点领域改革。加大“放管服”改革力度，持续营造法治化国际化便利化营商环境。深化行政审批制度改革，落实政府部门权责清单，精简创业创新、公共服务等领域行政许可事项，清理规范行政审批中介服务，推进行政审批标准化；开展“减证便民”专项行动，全面清理精简各类证明和手续。主动承接自贸试验区可复制、可推广的新一批试点经验。深化商事制度改革，推进“证照分离”“多证合一”，完善企业变更、注销程序，加快市场准入和退出便利化。完善预算管理体系，加强财政预算支出进度管理；加快财政事权和支出责任划分改革，优化市、区财政管理。推进环保、司法、审计、乡镇体制和供销社综合改革。深化金融改革，支持商业银行创新信贷产品和灵活还款付息方式，降低中小微企业融资成本；支持大中型骨干民营企业发起设立民营银行，鼓励有条件的县（市）开展“政银保”试点；开展农村普惠金融“村村通”试点，拓展“三农”综合保险新险种；推动国有、民营企业改制上市，支持在新三板、区域股权交易市场挂牌上市融资；规范发展互联网金融。引入第三方开展企业信用等级评价，加强诚信体系建设。坚持有进有退深化国企改革，支持湛化集团、汽运集团、包材公司等企业引入战略合作者，支持基投集团等4个投资公司提升投融资发展能力。明晰出资人权责清单，推进国有资产资本化，完善国有资产管理体制。

　　务实推进开放合作。加快建设“一带一路”海上合作战略支点城市。推进北部湾城市群一体化，重点推进社会保障、卫计医疗、住房公积金和旅游互联互通，办好首届北部湾城运会，加快建设粤桂北部湾经济合作区。融入琼州海峡经济带，深化徐闻县与海口市经贸合作，积极争取国家支持，共建海峡城市；联合开通海峡南北客运快船，完成港航资源整合，推进琼州海峡港航一体化，发展壮大海峡经济。推动与珠三角交通快速连接，主动承接珠三角产业转移，深化与港澳台经贸合作。推进与东盟等“海丝”沿线国家城市缔结友好关系，支持企业走向东盟，建设奋勇高新区东盟产业园，全面提升与东盟合作水平。

　　（八）坚持绿色发展，提高生态文明建设水平。深入推进“三个城市”建设。落实脚印城市建设八大行动计划，建成金沙湾风雨走廊；动工建设人民大道鼎盛路口立交；新增200辆新能源公交车、新开通公交线路6条；建成公交智能互动式电子站牌，申报建设公交都市。编制完成海绵城市专项规划，加快海绵工程项目建设。全面推广金沙湾示范区经验，全市实施生活垃圾分类，加快建设循环城市；落实国家循环经济示范城市建设任务，支持产业园区创建省级循环化改造示范园。树立节约集约、循环利用的资源观，彻底清查、充分利用批而未供、闲置存量土地，拓宽用地空间，提高用地效率。继续推进赤坎金沙湾“三个城市”样板区建设。

　　加大生态环境保护力度。推进实施蓝天保护、碧水重现、固废治理、绿化提升、宁静再造和市容环境六大工程。强化约束性指标管理，完成节能减排降碳目标任务。深化空气质量达标治理，推广使用清洁能源，全面淘汰黄标车。依法取缔不符合国家产业政策“十小”生产企业及生产项目，建设市工业固体废物处理中心。全面落实“河长制”，完成43个治水项目，加快推进主城区治水，集中整治城市建成区黑臭水体，开工建设屋山净化水质厂，如期实现主城区三年污水变清水目标。加大饮用水源保护力度，推进九洲江-鹤地水库流域和小东江、鉴江、南渡河环境综合整治。认真落实中央环保督查反映案件整改处置工作，全力解决环保突出问题。加强生态公共服务供给，建成东菊和东海岛生态公园，改造升级霞山儿童公园和瑞云湖公园，建设麻章体育公园和东山垌滨湖湿地。推进美丽海湾、生态岛礁和美丽海岸建设，有效保护湛江湾、海洋野生动物和候鸟资源，新增20个森林、湿地公园。扎实推进城乡人居环境整治，完成生活垃圾处理“一县一场”项目建设，新增绿化美化乡村192条。

　　（九）牢记以人民为中心，全力保障和改善社会民生。全力落实省十件民生实事，提高底线民生、扶贫济困、教育、基层医疗保障水平，促进就业创业，改善群众生产生活条件，加强生态保护，提高防灾减灾、基层公共服务和公共安全水平。办成市十件民生实事。完成森林公园升级改造；建成赤坎世贸、观海路和霞山国贸三座人行天桥；完成霞山观海路南段升级改造；建成1000套保障房；市区新增1万个优质学位；完成湛江中心人民医院整体搬迁；建成8间二甲乡镇医院和500间公建民营村级卫生站；实施15间镇级敬老院改扩建，新增床位1500个；建成市残疾人康复中心；完成赤坎区“一门式、一网式”政务服务模式改革试点。实施市十大民心工程。上半年动工建设西厅水厂，确保2018年上半年投入使用；启动人民大道等道路快速化改造；综合整治主城区22条河涌；动工建设湛江一中新校区；筹建广东医科大学新校区；基本完成市第一中医医院搬迁；动工建设市妇幼保健院海东新院；改造建设市公共卫生医院；改造人民大道南等9个地段人行道；实施中心镇通快速路工程，完成县道路面改造50公里、乡道升级改造100公里，建设农村硬底化公路500公里。

　　落实民生保障。全面落实“六个精准”“五个一批”要求，扎实开展脱贫攻坚，确保完成年度脱贫任务。坚持就业优先，新增城镇就业7.5万人，失业人员再就业3万人。完成机关事业单位养老保险制度改革。提高城乡居保基础养老金，落实征地农民养老保障待遇。提高困难群体大病保险标准，扩大异地联网结算定点医院范围。推动城镇居家养老服务全覆盖，力争每千名老人拥有30张以上养老床位。完善社会救助制度，筹建市救助管理站。

　　发展社会事业。创建5个以上“广东省推进教育现代化先进县（市、区）”。深化教育综合改革，推进普通高中、中职学校集中县城办学；撤并市区规模偏小的中职学校，腾出空间发展义务、学前教育，坚持学位资源与住宅小区规划、建设、使用“三同步”，持续增加中小学优质学位。推进岭师附中新校区和东海中学建设。推动省市共建地方本科高校，建设教育城二期，吸引更多高校、科研院所进驻，加快市高技能人才公共实训基地、市二技新校区建设，支持岭南师范学院新校区建设。支持海大寸金学院、广东文理职业学院提高办学水平。加快建设卫生强市，深化城市公立医院改革，探索组建医疗集团；大力发展中医药事业，实现县有中医院、镇有中医馆目标，继续评选名中医，改善名中医工作待遇；深化县镇医疗卫生服务管理一体化改革，加快县以下医疗卫生机构升级达标，鼓励符合条件的医师开设诊所，推行家庭医生签约服务，完善分级诊疗制度，县域内住院率达到90%左右。建成市120数字化信息指挥中心平台。办好第13届市运会、第4届环雷州半岛骑行活动。加大力度购买公共文化服务，推动文化设施向乡镇农村、城市社区倾斜，提高公共文化服务供给能力。重视保护城市历史文化风貌，开展自然村落历史人文普查和南粤古驿道保护利用。鼓励社会力量兴办文化展馆事业。编修百部地方志丛书。推进建设市档案馆二期。引入市场主体参与村级公共服务中心建设管理，推进创建基本公共服务均等化示范村。落实计划生育基本国策，促进人口均衡发展。加强军地沟通协调，推进军地深度融合发展，推动国防动员和后备力量建设。统筹推进统计、外事、侨务、港澳、对台、民族、宗教、气象、地震、防灾减灾、人防、打私、粮食、妇女儿童、残疾人等工作。

　　创新社会治理。坚决维护政治安全和社会稳定，加大治安突出问题整治力度，保持打击严重刑事犯罪高压态势，深化公安改革，加强公安“四项建设”，建立健全社会治安防控体系，切实提高社会治理社会化、法治化、智能化和专业化水平。持续开展平安创建，推动建设无毒村庄（社区），净化社会治安环境。加强“七五”法治宣传教育，落实法律援助工作。强化信访责任制，有效化解越级访、重复访和集体访。严格落实安全生产“党政同责、一岗双责、齐抓共管、失职追责”和企业主体责任，全面整治风险点，管控危险源，深化道路交通、危化品、建筑施工和人员密集场所等领域安全专项整治。坚持消防设施与其他市政设施同步规划、同步建设，加快县（市、区）消防站建设。

　　各位代表！打铁还需自身硬。完成目标任务，加快湛江振兴，建设清廉、务实、高效政府是关键。我们必须认真贯彻党的十八届六中全会精神，坚决把全面从严治党要求贯穿政府工作全过程，坚持以严的纪律、严的标准、严的举措建设勤廉双优政府。

　　第一，讲纪律，从严治政。坚持把纪律挺在前面，严守党的政治纪律和政治规矩，全面落实《关于新形势下党内政治生活的若干准则》和《中国共产党党内监督条例》规定要求，牢固树立“四个意识”，严肃规范党内政治生活，自觉在思想上政治上行动上始终同以习近平同志为核心的党中央保持高度一致。坚持党内监督和外部监督相结合，运用监督执纪“四种形态”强化党内监督，自觉接受人大依法监督、政协民主监督和司法监督、社会监督。加强审计监督，落实公共资金、国有资产、国有资源和领导干部经济责任审计全覆盖。推动构建亲而又清的新型政商关系，加强党风廉政建设，注重源头防腐，持续纠正“四风”，加大对专项资金特别是扶贫惠农资金的监管，严查发生在群众身边的腐败问题。

　　第二，讲法治，依法行政。坚持民主集中制，实行重大决策事项目录管理，规范“三重一大”集体决策行为。落实《法治政府建设实施纲要》，完善政府法律顾问制度；严格行政执法，落实行政执法责任制、执法全程记录和重大执法决定法制审核制度。坚持以百姓感受为标尺，改善政务服务环境，重点推进线上线下互动，优化审批流程，推动各级行政服务中心转型发展，打造便民高效的网上办事大厅。健全主动公开目录，推进政府决策、执行、管理、服务和结果“五公开”，引入第三方完善政府自媒体，推动政务更加阳光透明。

　　第三，讲奉献，实干勤政。坚持把强化落实、有效执行作为政府工作的生命线，严肃整治庸政懒政怠政行为。强化督查督办，构建“大督查”工作格局，确保政令“抵达终点”。坚持信任激励和严格监督相结合，开展机关绩效管理考核。健全容错纠错机制，鼓励干部改革创新、敢于担当、勇于负责、积极作为。全体公务员务必坚持不忘初心、继续前进，在勤勉实干、振兴湛江的过程中实现理想抱负、成就出彩人生。

　　各位代表！事业靠打拼，发展凭实干。未来五年，我们将兑现承诺、不负厚望，全面建成小康社会，加快建设环北部湾中心城市。面对光荣而艰巨的发展重任，我们没有选择、没有退路，只有把责任放在心上，扛在肩上，落实在行动上，实干大干，拼搏前行。让我们紧密团结在以习近平同志为核心的党中央周围，在中共湛江市委的坚强领导下，提振干事创业的精气神，焕发砥砺奋进的昂扬斗志，阔步走向充满希望的美好明天！
